# Neuregulin4 Acts on Hypothalamic ErBb4 to Excite Oxytocin Neurons and Preserve Metabolic Homeostasis

**DOI:** 10.1002/advs.202204824

**Published:** 2023-04-14

**Authors:** Yi Zhang, Yangyang Zhu, Jinghui Wang, Li Jin, Mingwei Guo, Liwei Chen, Lina Zhang, Yangyang Li, Baocheng Wan, Rong Zhang, Weiping Jia, Cheng Hu

**Affiliations:** ^1^ Shanghai Diabetes Institute Shanghai Key Laboratory of Diabetes Mellitus Shanghai Clinical Centre for Diabetes Shanghai Sixth People's Hospital Affiliated to Shanghai Jiao Tong University School of Medicine Shanghai 200233 P. R. China; ^2^ Institute for Metabolic Disease Fengxian Central Hospital Affiliated to Southern Medical University Shanghai 201449 P. R. China; ^3^ Department of Endocrinology Affiliated Hospital of Nantong University Nantong 226001 P. R. China; ^4^ Shanghai Key Laboratory of Regulatory Biology Institute of Biomedical Sciences and School of Life Sciences East China Normal University Shanghai 200241 P. R. China

**Keywords:** ErbB4, hypothalamus, neuregulin4, obesity, oxytocin neuron

## Abstract

Neuregulin 4 (Nrg4) is an adipose tissue‐enriched secreted factor that modulates glucose and lipid metabolism. Nrg4 is closely associated with obesity and preserves diet‐induced metabolic disorders. However, the specific mechanisms via which Nrg4 regulates metabolic homeostasis remain incompletely understood. Here, this work finds that the Nrg4 receptor, ErbB4, is highly expressed in the hypothalamus, and the phosphorylation of hypothalamic ErbB4 is reduced in diet‐induced obesity (DIO) mice. Peripheral Nrg4 can act on ErbB4 via blood circulation and excite neurons in the paraventricular nucleus of hypothalamus (PVN). Central administration of recombinant Nrg4 protein (rNrg4) reduces obesity and related metabolic disorders by influencing energy expenditure and intake. Overexpression of ErbB4 in the PVN protects against obesity, whereas its knock down in oxytocin (Oxt) neuron accelerates obesity. Furthermore, Nrg4‐ErbB4 signaling excites Oxt release, and ablation of Oxt neuron considerably attenuates the effect of Nrg4 on energy balance. These data suggest that the hypothalamus is a key target of Nrg4, which partially explains the multifaceted roles of Nrg4 in metabolism.

## Graphical abstract

Nrg4 secreted by adipose tissues can act on the ErbB4 receptor of oxytocin neurons in the hypothalamus and promote release of oxytocin peptide. Central administration of rNrg4 ameliorates dietary obesity by reducing appetite and increasing energy expenditure. This work highlights central Nrg4‐ErbB4 signaling as a potential therapeutic strategy for the treatment of obesity and related diseases.

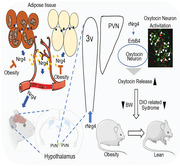

## 1 Introduction

Obesity is caused by excessive accumulation of body fat due to an imbalance between energy intake and consumption^[^ ^1^ ^]^ and substantially increases the risk of metabolic diseases,^[^ ^2^ ^]^ cardiovascular diseases,^[^ ^3^ ^]^ and cancers.^[^ ^4^ ^]^ Obesity is a topical field of research due to its high prevalence and detrimental effects. However, current research on the etiology and innovation in treatment methods remains insufficient.

Adipose tissue is a key “fuel” reservoir in the body and regulates various physiological processes through adipokines, including appetite production, fat distribution, insulin sensitivity changes, and inflammatory chemotactic responses.^[^ ^5^ ^]^ Adipokines are widely used tools for treating obesity due to their relative stability and ease of supply.^[^ ^6^ ^]^ Neuregulin 4 (Nrg4), a new type of adipokines mainly produced in white and brown adipose tissues, is closely associated with energy balance and glycolipid metabolism. Nrg4 preserves metabolic homeostasis by attenuating hepatic lipogenic signaling,^[^ ^7^ ^]^ promoting adipocyte thermogenesis,^[^ ^8^ ^]^ and increasing energy expenditure.^[^ ^9^ ^]^ Of note, the Nrg4 receptor Erb‐B2 Receptor Tyrosine Kinase 4 (ErbB4) is sparsely expressed in peripheral metabolic organs, such as adipocytes, liver, muscles, and islet cells. Hence, the specific mechanisms of Nrg4‐ErbB4 signaling in energy homeostasis remain to be explored.

ErbB4, a receptor tyrosine kinase widely expressed in the central nervous system (CNS), plays an important role in neuronal proliferation, migration, differentiation, neurite outgrowth and axon guidance, as well as synapse formation and plasticity.^[^ ^10^ ^]^ ErbB4 is a susceptibility factor for schizophrenia. Human and mouse experiments have confirmed that mutations in ErbB4 lead to schizophrenia.^[^ ^11^ ^]^ Interestingly, genetic association showed that the ErbB4 mutation in the population was closely related to both schizophrenia and diabetes,^[^ ^11^, ^12^ ^]^ suggesting that ErbB4 may be the common pathogenic mechanism leading to schizophrenia and diabetes. ErbB4 whole‐body deletion in mice led to the development of obesity and related metabolic dysfunction,^[^ ^13^ ^]^ but the specific mechanism by which ErbB4 regulates metabolism is still unclear. Interestingly, ErbB4 is abundantly expressed in the hypothalamus and rarely expressed in peripheral metabolic organs. Hence, ErbB4 may regulate metabolic homeostasis through the hypothalamus.

The CNS, especially the hypothalamus, is well‐established as the central regulator of energy metabolism.^[^ ^14^ ^]^ Many peripheral hormones, such as leptin, insulin, and ghrelin, regulate glucose and lipid metabolism by acting on the hypothalamus.^[^ ^15^ ^]^ Several hypothalamic nuclei, including the paraventricular nucleus of the hypothalamus (PVN) and arcuate nucleus (Arc), participate in energy balance.^[^ ^16^ ^]^ The PVN is a key hub that receives upstream signaling molecules and regulates energy intake and expenditure.^[^ ^17^ ^]^ In recent years, the role of PVN oxytocin (Oxt) expressing neurons in regulating energy balance has been gaining recognition.^[^ ^18^ ^]^ Although Nrg4 is sparsely expressed in the brain, its receptor, ErbB4, is abundantly and specifically expressed in the hypothalamus, especially Oxt neurons in PVN.^[^ ^19^ ^]^ The released N‐terminal EGFL polypeptide segment of Nrg4 is only 14 kDa and is fully capable of crossing the blood‐brain barrier. Hence, the adipose‐derived endocrine factor Nrg4 may regulate glucose and lipid metabolism by acting on the hypothalamus.

In this study, we will focus on the central effects of Nrg4 on energy metabolism by targeting ErbB4 in the hypothalamic Oxt neurons. Through this research, we aim to partially explain the specific mechanisms of Nrg4 in metabolic balance and provide novel insights into obesity treatment.

## 2 Results

### 2.1 Impaired ErbB4 Signaling in the Hypothalamus of HFD‐Fed Mice

**Figure 1:**
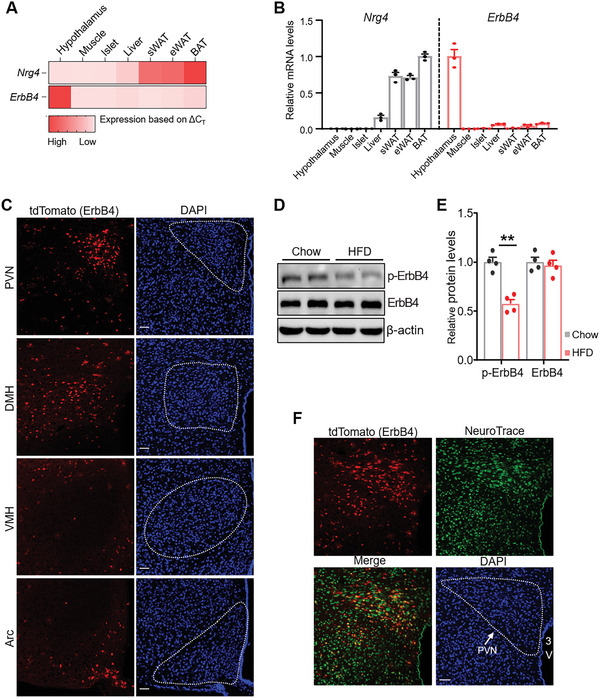
Impaired ErbB4 signaling in the hypothalamus of HFD‐fed mice. A,B) Metabolic tissue‐specific expression levels of Nrg4 and ErbB4 were measured in lean C57BL/6J male mice and are presented as ΔCT values (*n* = 3 for each group). C) tdTomato of ErbB4‐CreER::Ai14 mice showing ErbB4 expression in the PVN, DMH, VMH, and Arc of the hypothalamus. Cell nuclei were counterstained with DAPI. Scale bar, 50 µm. D) Western blot (WB) analysis of ErbB4 and pErbB4 in the hypothalamus of chow‐ or HFD‐fed mice. *β*‐actin was used as loading control. E) Quantification of the Western blots for ErbB4 and pErbB4. *n* = 4 for each group. F) Fluorescent Nissl staining (Neuro Trace, neurons, green) in PVN of ErbB4‐CreER::Ai14 mice. 3 V, third ventricle. Cell nuclei were counterstained with DAPI. Scale bar, 50 µm. Data are presented as mean ± SEM; **p < 0.01, two‐tailed Student's *t*‐test (E).

First, we systematically detected the expression of Nrg4 and its receptor, ErbB4, in metabolic tissues in mice. Quantitative reverse transcription PCR (qRT‐PCR) results revealed that Nrg4 was expressed in a limited number of adult tissues, such as brown adipose tissues, and had negligible expression in the brain (**Figure** **1**A,B). However, ErbB4 was abundantly expressed in the hypothalamus, which is the center of glucose and lipid metabolism, rather than in other metabolic tissues (Figure 1A,B). Moreover, we used the *ErbB4‐CreER::Ai14* mice, in which ErbB4^+^ cells were visualized by tdTomato to show the ErbB4 expression in the hypothalamus, and found that ErbB4 was expressed in the PVN, dorsomedial hypothalamus, and Arc of hypothalamic nuclei (Figure 1C). We then detected the changes of hypothalamic ErbB4 in the diet induced obesity (DIO) mice. Western blot (WB) analysis revealed that hypothalamic ErbB4 level was similar among DIO mice, suggesting that high‐fat diet (HFD) feeding had little effect on ErbB4 protein level (Figure 1D,E). Interestingly, ErbB4 tyrosine phosphorylation (pErbB4) was markedly reduced in the hypothalamus (Figure 1D,E). The reduced hypothalamic pErbB4 in obesity may be due to the decreased Nrg4 secreted by adipocytes. Nrg4 expression of adipose tissue is reduced in HFD‐fed mice,^[^ ^7^, ^8^, ^9^ ^]^ possibly leading to decreased levels in the hypothalamus and reduced phosphorylation of ErbB4. We then performed neuron trace staining on brain sections of ErbB4‐CreER::Ai14 mice to determine whether ErbB4 was expressed in PVN neurons. Indeed, we observed that ErbB4 was expressed in most PVN neurons (Figure 1F). Taken together, these results indicated that ErbB4 may mediate the function of PVN on metabolic syndrome.

### 2.2 Peripheral Nrg4 Can Act on ErbB4 and Excite Neurons in PVN

**Figure 2:**
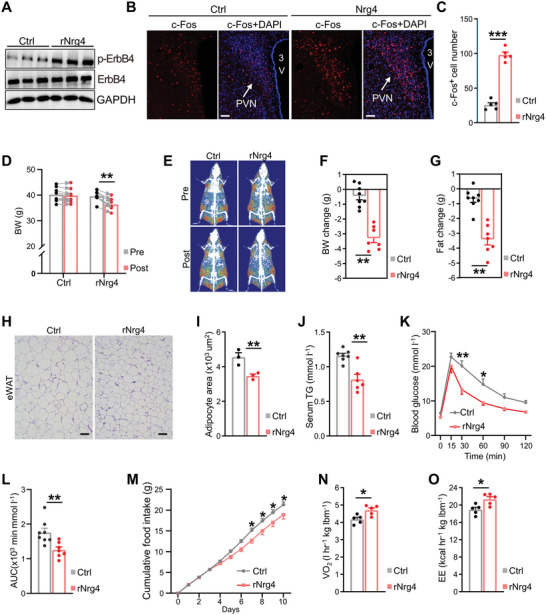
Nrg4 targets the brain to preserve metabolic homeostasis A) WB analysis of pErbB4 and ErbB4 from hypothalamic lysates of mice treated with Ctrl or rNrg4 (500 µg kg^−1^) for 30 min. B) Immunofluorescence staining of c‐Fos in the PVN of mice intraperitoneally injected with rNrg4 (500 µg kg^−1^) or control for 2 h. Scale bar, 100 µm. C) Numbers of c‐Fos^+^ cells in the PVN (*n* = 5 for each group). D–G) DIO mice receiving daily central administration of rNrg4 or Ctrl for 10 days. D) Body weight, E) representative DEXA images, F) body weight changes, and G) fat mass changes. Ctrl, 0.9% NaCl‐injected mice (*n* = 8 for Ctrl, *n* = 7 for rNrg4). H,I) H&E staining (H) and mean area of adipocytes (I) in epididymal white adipose tissue (eWAT) from mice administered Ctrl or rNrg4 (*n* = 3 for each group). Scale bar, 100 µm. J) Serum triglyceride (TG) levels of mice administered Ctrl or rNrg4 (*n* = 7 for Ctrl, *n* = 6 for rNrg4). K,L) Glucose tolerance test (GTT, K) and AUC of GTT(L) of mice injected with Ctrl or rNrg4 (*n* = 8 for Ctrl, *n* = 7 for rNrg4). M) Cumulative food intake of mice injected with Ctrl or rNrg4 (*n* = 8 for Ctrl, *n* = 7 for rNrg4). N,O) HFD‐fed mice were i.c.v. injected saline or rNrg4. Oxygen consumption (VO_2_, N) and Energy expenditure (EE, O) for 8 h post treatment are shown (*n* = 5 for each group). lbm, lean body mass. Data are presented as mean ± SEM. *p < 0.05, **p < 0.01, ***p < 0.001, two‐tailed Student's *t*‐test (C,F,G,I,J,L,N,O); two‐way analysis of variance (ANOVA) with Bonferroni's post hoc test (D,K,M).

The small‐molecule properties of Nrg4 secreted protein and high expression of ErbB4 in the hypothalamus suggested that fat‐enriched Nrg4 may act on the hypothalamus through the blood circulation. We purified a Nrg4 recombinant protein (rNrg4) to demonstrate our speculation. After verifying the function of the rNrg4 (Figure S1, Supporting Information), we intraperitoneally injected rNrg4 into mice. WB analysis of His‐tag showed that rNrg4 was indeed detected in the hypothalamus (Figure S2A, Supporting Information). We also found that the p‐ErbB4 was obviously enhanced in the hypothalamus, indicating that Nrg4 can act on the hypothalamus through the blood circulation (**Figure** **2**A). Moreover, we found that c‐Fos^+^ cells were markedly increased in the PVN and slightly increased in the Arc, but no significant differences were observed in other hypothalamic nuclei (Figure 2B,C, Figure S2B,C, Supporting Information). As the PVN plays a key role in obesity and related metabolic syndrome, the findings suggest that Nrg4‐ErbB4 regulates obesity partially via hypothalamic PVN.

### 2.3 Nrg4 Preserves Metabolic Homeostasis via the CNS

We hypothesized that Ngr4 preserves metabolic homeostasis partially via the hypothalamus. To test this, we performed lateral ventricle cannulation surgery on mice fed a HFD for 12 weeks. We found that intracerebroventricular (i.c.v.) administration of rNrg4 at doses ≥500 ng per mouse extensively increased c‐Fos^+^ in PVN and reduced mice food intake (Figure S3, Supporting Information). Hence, we chose the dose of 500 ng per mouse to treat mice unless otherwise noted. We then administered all mice a daily i.c.v. injection of saline (Ctrl) or 500 ng of rNrg4 directly into the lateral ventricle. Mice administered rNrg4 into the brain gained considerably less body weight compared to controls (Figure 2D–F). Body composition analysis revealed that this effect was predominantly due to a decrease in fat mass rather than lean mass (Figure 2G, Figure S4C, Supporting Information). Consistent with this, epididymal white adipose tissue (eWAT) was markedly smaller than that in controls (Figure 2H,I). Liver steatosis was notably reduced due to rNrg4 treatment (Figure S2B, Supporting Information). Serum triglyceride (TG) levels were lower in Nrg4‐treated mice than in control mice (Figure 2J). Since obesity is closely associated with blood glucose, we measured glucose tolerance in central rNrg4‐infused mice (Figure 2K,L). Glucose tolerance was improved compared to that in control mice. To elucidate the causes of central Nrg4‐induced reduction in dietary obesity, we measured food intake and energy expenditure in these mice. We observed that central rNrg4‐treated mice consumed much less HFD, although the difference was modest (Figure 2M). Indirect calorimetry analysis revealed that central rNrg4 injection promoted oxygen consumption (Figure 2N) and heat production (Figure 2O). These results demonstrate that adipocytes secreting Nrg4 partially protects against DIO via the CNS.

### 2.4 PVN^ErbB4^ Neurons Mediate the Effects of Nrg4 on Regulating Energy Balance

**Figure 3:**
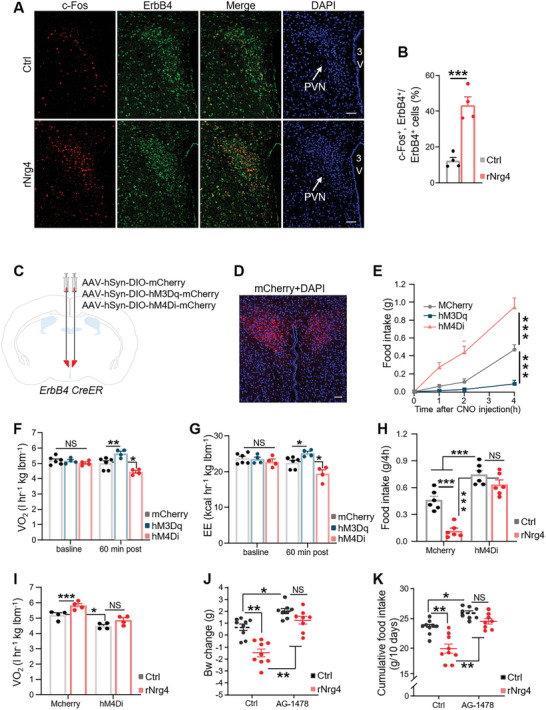
PVN ErbB4 neurons mediate Nrg4's effect on energy balance. A) Double immunostaining of ErbB4 (green) and c‐Fos (red) in the PVN of control‐ or Nrg4‐administered mice. Cell nuclei were counter‐stained with DAPI. Scale bar, 50 µm. B) Percentage of ErbB4+ cells expressing c‐Fos in the PVN of Ctrl or rNrg4‐administered mice (*n* = 4 for each group). C) A diagram depicting bilateral injection of AAV‐hSyn‐DIO‐mCherry, AAV‐hSyn‐DIO‐hM3Dq‐mCherry, and AAV‐hSyn‐DIO‐hM4Di‐mCherry into the PVN of ErbB4 CreER mice. D) Representative image showing mCherry expression in the PVN. The section was counter‐stained with DAPI. Scale bar: 50 µm. E) HFD‐fed ErbB4 CreER mice expressed mCherry, hM3Dq‐mCherry, hM4Di‐mCherry in PVN were briefly fasted and treated with CNO immediately before light‐off. Food intake during the following 1, 2, and 4 h were shown (*n* = 7 for mCherry, hM3Dq‐mCherry mice, *n* = 6 for hM4Di‐mCherry). F,G) VO_2_ (F) and EE (G) average during 1 h period before and after CNO injection in mCherry, hM3Dq‐mCherry and hM4Di‐mCherry mice under HFD feeding for 2 weeks (*n* = 7 for mCherry mice, *n* = 6 for hM3Dq‐mCherry and hM4Di‐mCherry). H) HFD‐fed ErbB4 CreER mice expressed mCherry and hM4Di‐mCherry in PVN were briefly fasted and treated with CNO and saline or rNrg4. Food intake for 4 h post treatment was shown (*n* = 6 for each group). lbm, lean body mass. I) HFD‐fed ErbB4 CreER mice expressed mCherry and hM4Di‐mCherry in PVN were treated with saline or rNrg4. VO2 for 4 h post treatment was shown (*n* = 4 for each group). lbm, lean body mass. J,K) HFD‐fed mice were centrally administered control or 20 nmol of the ErbB4 antagonist, AG‐1478. After 1 h, mice received i.c.v. injections of control or 500 ng of rNrg4 for 10 consecutive days. Body weight changes (J) and cumulative food intake (K) were measured (*n* = 9 for Ctrl, *n* = 8 for AG‐1478). Data are presented as mean ± SEM; *p < 0.05, **p < 0.01, two‐tailed Student's *t*‐test (B); two‐way analysis of variance (ANOVA) with Bonferroni's posthoc test (E–K).

After demonstrating the specificity of ErbB4 antibody (Figure S5, Supporting Information), we further observed that rNrg4 activated ErbB4^+^ cells in the PVN, as demonstrated by c‐Fos and ErbB4 double immunostaining (**Figure** **3**A,B). These results indicate that ErbB4 in PVN neurons may mediate the effects of Nrg4 on energy balance. We then tested whether PVN ErbB4 neurons regulated body weight balance. We manipulated the activity of PVN ErbB4 neurons using designer receptors exclusively activated by designer drugs (DREADDs) and their ligand clozapine N‐oxide (CNO).^[^ ^20^ ^]^ We expressed stimulatory DREADD hM3Dq, inhibitory DREADD hM4Di, or mCherry in PVN ErbB4 neurons by bilaterally injected with Cre‐dependent AAV‐hSyn‐DIO‐hM4Di‐mCherry, AAV‐hSyn‐DIO‐hM3Dq‐mCherry or AAV‐hSyn‐DIO‐mCherry into PVN of *ErbB4 CreER* mice(Figure 3C, Figure S6A, Supporting Information).^[^ ^21^ ^]^ Then, the expression of injected AAV viruses in the PVN ErbB4 neurons was induced by treating mice with tamoxifen (Figure 3D, Figure S6B, Supporting Information). We found that treatment of CNO greatly influenced food intake and energy expenditure of HFD‐fed ErbB4 CreER mice expressing hM3Dq‐mCherry and hM4Di‐mCherry (Figure 3E–G). More importantly, we found that inhibition of PVN ErbB4 extensively abolished the effects of Nrg4 on appetite (Figure 3I) and energy expenditure (Figure 3J, Figure S6B, Supporting Information). Taken together, these chemogenetic results indicated that ErbB4 in PVN neurons mediate the effects of Nrg4 on energy balance. To further confirm whether ErbB4 mediated the anti‐obesity effects of central Nrg4, HFD‐fed mice received i.c.v. administration of an ErbB4 antagonist, AG‐1478, followed by central injection of rNrg4. AG‐1478 markedly attenuated the effects of rNrg4 on food intake and body weight, indicating that ErbB4 in the brain is a key mediator of central Nrg4 in energy metabolism (Figure 3J,K). In agreement with this, AG‐1478 inhibited the increased number of c‐Fos^+^ cells in the PVN induced by rNrg4 (Figure S6D,E, Supporting Information).

### 2.5 Overexpression of ErbB4 in PVN Protects Against DIO

**Figure 4:**
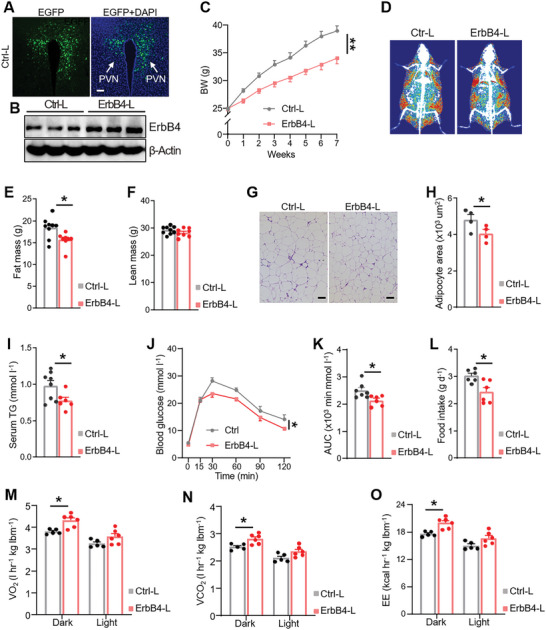
Overexpression of ErbB4 in the PVN protects against DIO. A) EGFP expression (green) after injection of the control lentivirus (Ctrl‐L) into the PVN. Cell nuclei were counterstained with DAPI (blue). Scale bar, 100 µm. B) Western blot analysis of ErbB4 expression in PVN lysates of mice injected with Ctrl‐L and ErbB4‐L viruses (*n* = 3 for each group). C–F) Body weights(C), DEXA images(D), fat mass (E), and lean mass(F) of mice injected with Ctrl‐L or ErbB4‐L virus into the PVN under a HFD diet (*n* = 9 for Ctrl‐L, *n* = 8 for ErbB4‐L). G,H) H&E staining (G) and mean area (H) of adipocytes of eWAT of mice fed a HFD for 20 weeks (*n* = 4 for each group). Scale bar, 100 µm. I) Serum TG levels of mice fed a HFD for 20 weeks. (*n* = 8 for Ctrl‐L, *n* = 6 for ErbB4‐L). J,K) GTT (J) and AUC of GTT (K) of mice fed a HFD for 8 weeks (*n* = 8 for Ctrl‐L, *n* = 7 for ErbB4‐L). L) Daily food intake of Ctrl‐L and ErbB4‐L mice (*n* = 7 for each group). M,N) Mice were intra‐PVN injected with Ctrl‐L and ErbB4‐L viruses. VO_2_ (M), carbon dioxide (VCO_2_, N), and O) EE of Ctrl‐L and ErbB4‐L mice fed a HFD for 1 week. (*n* = 5 for Ctrl‐L, n = 6 for ErbB4‐L). Data are presented as mean ± SEM; *p < 0.05, **p < 0.01, two‐tailed Student's *t*‐test (E,H,I,K,L); two‐way analysis of variance (ANOVA) with Bonferroni's post hoc test (C,J,M–O).

To test whether ErbB4 expression in the PVN protects against DIO, we generated an ErbB4‐expressing lentiviral plasmid and enhanced green fluorescent protein (EGFP)‐expressing control vector, referred to as ErbB4‐L and Ctrl‐L, respectively. We delivered these lentiviruses into the PVN of chow‐fed C57BL/6 mice (Figure S7A, Supporting Information) and confirmed the success of surgery and effectiveness of lentiviruses (**Figure** **4**A,B). Overexpression of ErbB4 in the PVN resulted in less body weight gain under HFD feeding compared to that in controls (Figure 4C), which was predominantly due to a smaller increase in fat mass (Figure 4D–H; Figure S7B, Supporting Information). In addition, serum TG levels and glucose tolerance were improved in ErbB4‐L mice (Figure 4I–K). Further indirect calorimetry analysis revealed that these mice consumed less food, more oxygen, and produced more carbon dioxide and heat (Figure 4L–N). Moreover, overexpression of ErbB4 in the PVN enhanced the effects of central rNrg4 on energy metabolism. Central rNrg4 injections in ErbB4‐L mice resulted in lower body weight and food intake (Figure S7D,E, Supporting Information). Collectively, these results demonstrated that Nrg4 ameliorates diet‐induced metabolic dysfunction by targeting ErbB4 in the PVN.

### 2.6 Oxt Neurons Mediate the Effects of Nrg4 on Energy Metabolism in DIO Mice

**Figure 5:**
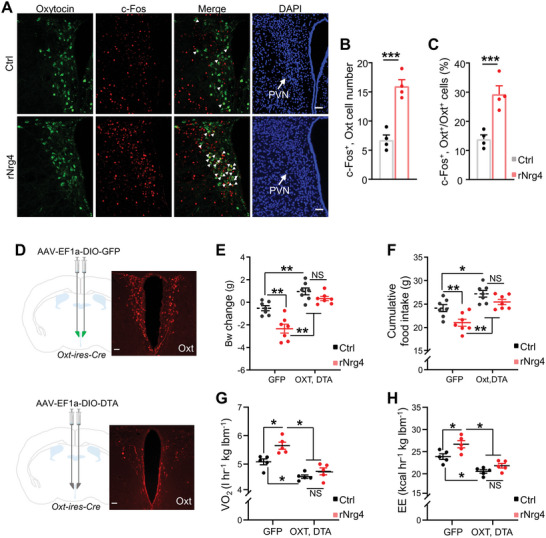
Blocking Oxt attenuates the effects of rNrg4 on energy balance. A) Double immunofluorescence staining of oxytocin (green) and c‐Fos (red) in the PVN of mice administered control or rNrg4. Cell nuclei were counterstained with DAPI (blue). Arrows indicate c‐Fos and Oxt co‐expressing cells. Scale bar, 50 µm. B,C) Number of c‐Fos+ and Oxt+ cells (B) and percentage of Oxt+ cells expressing c‐Fos (C) in the PVN (*n* = 4 for each group). D) Schematic representation of GFP and DTA virus injection sites with representative images of Oxt expression in the PVN. Scale bar, 100 µm. E,F) Adult male Oxt‐ires‐Cre mice were injected with AAV‐DIO‐GFP (Ctrl) or AAV‐Ef1a‐DIO‐DTA (DTA) viruses into the PVN and were fed a HFD for 4 weeks. The mice were centrally injected with saline or rNrg4 for 10 consecutive days. Body weight changes (E) and cumulative food intake (F) were measured (*n* = 7 for each group). G,H) Ctrl and Oxt neuron‐ablated mice were centrally injected with saline or rNrg4 immediately before dark light. VO_2_ (G) and EE (H) were then measured for 12 h in the dark light after injection (*n* = 5 for AAV‐DIO‐GFP, *n* = 6 for AAV‐DIO‐DTA). lbm, lean body mass. Data are presented as mean ± SEM; *p < 0.05, **p < 0.01, two‐tailed Student's *t*‐test (B,C); two‐way analysis of variance (ANOVA) with Bonferroni's correction (E–H).

The PVN contains several types of peptidergic neurons. Hence, we explored which type(s) of neurons in the PVN mediate the effects of Nrg4 on energy metabolism. Immunofluorescence staining of c‐Fos, Oxt, and arginine vasopressin (AVP) revealed that rNrg4 specifically activates Oxt neurons in the PVN (**Figure** **5**A–C; Figure S8A,B, Supporting Information). Since Oxt in the PVN is a key neuropeptide regulating energy balance, we speculated that Oxt neuron mediates the effects of Nrg4 on preservation of metabolic homeostasis. To test this, we ablated Oxt neurons by injecting Cre‐dependent DTA or GFP into the PVN of *Oxt‐ires‐Cre* mice (Figure 5D, Figure S8C–E, Supporting Information) followed by i.c.v. administration of rNrg4 or saline after 4 weeks of exposure to HFD. Oxt neuron ablation markedly attenuated the effects of rNrg4 on body weight (Figure 5E), food intake (Figure 5F), oxygen consumption (Figure 5G), and energy expenditure (Figure 5H). These results suggested that Oxt neurons are a key mediator of the effects of Nrg4 on energy metabolism.

### 2.7 Oxt Neuron‐Specific Knockdown of ErbB4 Aggravates Obesity

**Figure 6:**
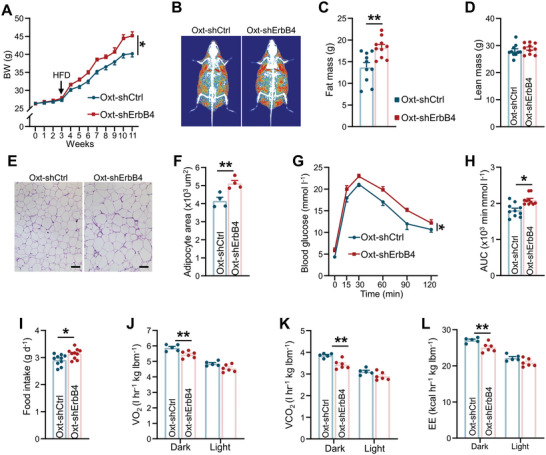
Oxt neuron‐specific knockdown of ErbB4 aggravates obesity. A) Body weights of mice injected with either Oxt‐shCtrl or Oxt‐shErbB4 virus into the PVN. Mice were fed a chow diet for the first 3 weeks after injection, and then mice were switched to a HFD (*n* = 10 per group). B–D) DEXA images (B), fat mass (C), and lean mass (D) of mice injected with either Oxt‐shCtrl or Oxt‐shErbB4 virus into the PVN under a HFD diet for 10 weeks (*n* = 10 for each group). E,F) Representative H&E staining images (E) and mean eWAT area (F) of Oxt‐shCtrl and Oxt‐shErbB4 mice (*n* = 4 for each group). Scale bar, 50 µm. G,H) GTT (G) and AUC of GTT (H) of mice fed a HFD for 12 weeks (*n* = 10 for each group). I) Average food intake of Oxt‐shCtrl or Oxt‐shErbB4 mice fed a HFD (*n* = 10 for each group). J–L) O_2_ consumption (J), CO_2_ production(K), and EE (L) of mice fed a HFD for 1 week. (*n* = 5 for Oxt‐shCtrl, *n* = 6 for Oxt‐shErbB4). Data are presented as mean ± SEM; *p < 0.05, **p < 0.01, two‐tailed Student's *t*‐test (C,D,F,H,I); two‐way analysis of variance (ANOVA) with Bonferroni's correction (A,G,J–L).

To further explore Oxt neuron‐specific roles of ErbB4 in DIO, *Oxt* gene promoter was used to generate Oxt neuron‐specific ErbB4‐targeted shRNA lentiviral expression plasmid (Oxt‐shErbB4) or mCherry as a control (Oxt‐shCtrl). We verified that the viruses indeed marked Oxt neuron and specifically inhibited ErbB4 expression in Oxt neurons by examining the PVN of virus‐injected mice (Figure S9A–C, Supporting Information). No significant differences were noted in body weight changes between the two groups after virus injection for 3 weeks under a chow diet (**Figure** **6**A). We then shifted the mice to a HFD and observed that Oxt‐shErbB4 mice gained considerably more body weight compared to Oxt‐shCtrl mice (Figure 6A,B). Body composition analysis revealed that body weight gain was predominantly due to an increase in fat mass rather than lean mass (Figure 6C,D). Consistent with these results, eWAT size was markedly larger than that of controls (Figure 6E,F). Moreover, HFD‐induced liver steatosis was significantly accelerated in Oxt‐shErbB4 mice (Figure S9D, Supporting Information). Oxt neuron‐specific knockdown of ErbB4 resulted in glucose intolerance (Figure 6G,H). Additionally, the appetite of Oxt‐shErbB4 mice was significantly increased, although the effect was subtle (Figure 6I). Further assessment revealed that Oxt‐shErbB4 mice consumed less oxygen and produced less carbon dioxide as well as energy expenditure before body changes (Figure 6J–L). Collectively, these data indicated that Oxt neuron‐specific knockdown of ErbB4 aggravates obesity.

### 2.8 Nrg4‐ErbB4 Signaling Preserves DIO Induced Metabolic Dysfunction via Regulating Oxt Release from the PVN

**Figure 7:**
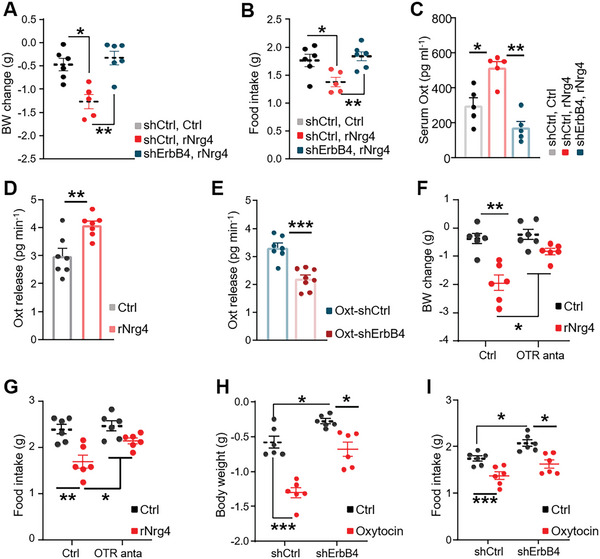
Nrg4‐ErbB4 signaling regulates Oxt release from the PVN. A,B) Oxt‐shCtrl or Oxt‐shErbB4 mice under a HFD feeding were treated with rNrg4 or saline; 24 h body weight (A) and food intake (B) were measured. (*n* = 5 for Oxt‐shCtrl and rNrg4; *n* = 6 for remaining groups). C) HFD‐fed mice were injected with Oxt‐shCtrl or Oxt‐shErbB4 virus into the PVN, treated with rNrg4 or saline for 7 consecutive days, and serum Oxt levels were measured. (*n* = 5 for Oxt‐shCtrl, *n* = 6 for Oxt‐shErbB4). D) Basal and rNrg4 (1 µg mL^−1^)‐elicited Oxt release in PVN slices of HFD‐fed mice (*n* = 5 for Ctrl, *n* = 6 for rNrg4). E) Basal Oxt release of PVN slices of HFD‐fed Oxt‐shCtrl or Oxt‐shErbB4 mice (*n* = 9 for Oxt‐ShCtrl, *n* = 8 for Oxt‐ShErbB4). F,G) HFD‐fed mice were centrally administered with control or 2 µg of an OXT antagonist, L‐372662. After 1 h, mice received daily i.c.v. injections of control or 500 ng of rNrg4. 24 h body weight changes (F) and cumulative food intakes (G) were measured (*n* = 7 for each group). H,I) HFD‐fed Oxt‐shCtrl and Oxt‐s hErbB4 mice were treated with control or Oxytocin, body weight changes (H) and food intake (I) were measured (*n* = 7 for each group). Data are presented as mean ± SEM; *p < 0.05, **p < 0.01, ***p < 0.001, two‐tailed Student's *t*‐test (D,E); one‐way analysis of variance (ANOVA) with Bonferroni's correction (A–C); two‐way analysis of variance (ANOVA) with Bonferroni's correction (F–I).

We found that ErbB4 knockdown in Oxt neurons markedly abolished the effects of rNrg4 on body weight (**Figure** **7**A) and food intake (Figure 7B). The effects of rNrg4 on energy balance were also attenuated (Figure S10A, Supporting Information). These results indicated that central Nrg4 exerts anti‐obesity function through ErbB4 in Oxt neurons. As impaired Oxt release from the PVN is involved in DIO pathogenesis, its blockage via gentic or pharmacologic interventions promoted body weight loss, while treatment of Oxt can effectively correct hyper‐appetite and obesity.^[^ ^22^ ^]^ We explored whether Nrg4‐ErbB4 signaling regulates Oxt release. We first examined serum Oxt levels in Oxt‐shErbB4 and Oxt‐shCtrl mice treated with either rNrg4 or saline, respectively. Indeed, Oxt levels were considerably increased after rNrg4 treatment, and Oxt neuron ErbB4 knockdown markedly abolished the effects of rNrg4 (Figure 7C). We then performed an Oxt release assay to verify these findings. PVN slices dissected from DIO mice were treated with rNrg4, which resulted in a notable increase in the ex vivo Oxt release rate (Figure 7D). Consistent with this, the ex vivo Oxt release rate in ErbB4‐L mice was markedly increased (Figure S10B, Supporting Information), and Oxt release was also notably attenuated in Oxt‐shErbB4 mice under a HFD (Figure 7E). The Oxt receptor (OTR) mediates the anti‐obesity effects of released Oxt from PVN.^[^ ^23^ ^]^ Hence, we then i.c.v. pre‐administered the OTR antagonist, L‐368899, before rNrg4 delivery to HFD‐fed mice. Consistent with the above results, pretreatment with L‐368899 abolished the central effects of rNrg4 in regulating energy homeostasis (Figure 7F,G). We last demonstrated the role of oxytocin in ErbB4‐mediated regulation of energy balance. We injected Oxt peptide directly to Oxt‐shCtrl or Oxt‐shErbB4 mice and found that Oxt could rescue obesity caused by ErbB4 knockdown in PVN neurons (Figure 7H,I). Hence, these results indicated that Nrg4‐ErbB4 signaling protected against DIO‐induced metabolic dysfunction by exciting Oxt release from PVN.

## 3 Discussion

Nrg4 belongs to the epidermal growth factor (EGF) family and is a new type of adipokine that is predominantly expressed in the adipose tissues.^[^ ^24^ ^]^ Nrg4 is hydrolyzed at serine 53 of the transmembrane segment, which releases a highly conserved terminal EGFL polypeptide that acts on target cells through endocrine mechanisms.^[^ ^25^ ^]^ Wang et al. reported that BAT controls liver lipogenesis through the secretion of the growth factor Nrg4.^[^ ^7^ ^]^ BAT‐secreted Nrg4 acts on ErbB3/ErbB4 signaling in hepatocytes, attenuates hepatic lipogenic signaling, and preserves glucose and lipid homeostasis in obesity. Furthermore, Nrg4 expression is substantially downregulated in mice and humans with obesity.^[^ ^9^, ^26^ ^]^ Comas et al. demonstrated that the expression of Nrg4 is a novel marker of beige adipocytes in human adipose tissue.^[^ ^27^ ^]^ Moreover, recent studies have demonstrated that Nrg4 stimulates browning of WAT,^[^ ^28^ ^]^ exacerbates BAT activity,^[^ ^29^ ^]^ enhances blood vessels of adipose tissue, increases neurite outgrowth through neuronal interactions in adipose tissues,^[^ ^26^ ^]^ and inhibits the development of hepatocellular carcinoma.^[^ ^30^ ^]^ Interestingly, the Nrg4 receptor ErbB4 is abundantly expressed in the hypothalamus.^[^ ^19^ ^]^ Zhang et al. found that Nrg1, another member of Nrg family, can reduce food intake and active hypothalamic POMC neurons.^[^ ^31^ ^]^ In this regard, Nrg4 may act on the hypothalamus to regulate glucose and lipid metabolism.

In the present study, we focused on the metabolic role of Nrg4 in the hypothalamus. We found that peripheral Nrg4 can enter the hypothalamus through the blood‐brain barrier and activate hypothalamic Oxt neurons. Moreover, central administration of rNrg4 ameliorated DIO and related dysfunction. We further demonstrated that Nrg4 targets ErbB4 expressed in PVN Oxt neurons to maintain metabolic homeostasis. Our findings provide deeper mechanistic understanding of the effects of Nrg4 on energy metabolism and suggest that Nrg4‐ErbB4 signaling mediates adipose‐brain crosstalk in regulating energy balance.

ErbB4 plays various roles in physiological and pathological states. Genetic studies have indicated that ErbB4 is closely associated with obesity and type 2 diabetes,^[^ ^11^, ^12^ ^]^ but its specific role in metabolic syndrome remains poorly understood. Here, we observed that ErbB4 was abundantly expressed in the hypothalamus and that phosphorylation of ErbB4 was reduced in diet induced obesity (DIO) mice. Moreover, we found that ErbB4 overexpression in the PVN protected against, whereas ErbB4 knockdown in PVN Oxt neurons accelerated, DIO‐induced metabolic dysfunction. We also demonstrated that ErbB4 in PVN neurons regulated Oxt release. In this study, the glucose tolerance was also regulated by ErbB4 expressed in Oxt neurons, which may be independent of obesity since we found that glucose tolerance changed considerably when mice were fed a HFD for 4 weeks, in which time the body weight changes slightly (Figure S10, Supporting Information). As Oxt directly stimulated insulin secretion^[^ ^32^ ^]^ and central Oxt treatment improved glucose tolerance in both DIO and STZ‐induced diabetic mice,^[^ ^22^, ^32^, ^33^ ^]^ the central Nrg4‐Erbb4 signaling may modulate glucose metabolism through its action on Oxt neurons. Taken together, our study reveals the specific cellular mechanisms via which ErbB4 regulates energy balance, highlighting ErbB4 in the PVN as a novel therapeutic target for obesity. ErbB4 is a susceptibility factor for schizophrenia, and a large number of studies have shown that impairment of ErbB4 signaling pathway contributes to schizophrenia.^[^ ^11a^ ^]^ Hence, ErbB4 is a common susceptibility gene for schizophrenia and diabetes, which partly explains why people with schizophrenia are more likely to develop diabetes.^[^ ^34^ ^]^

Oxt, a hypothalamic neuropeptide that induces parturition and lactation in females, is synthesized and released by neurons that are predominantly localized in the PVN and SON of the hypothalamus.^[^ ^35^ ^]^ Recent research has focused on the role of PVN Oxt in suppressing food intake and promoting energy intake.^[^ ^18^ ^]^ Oxt^[^ ^23a^ ^]^ or OTR deletion^[^ ^23b^ ^]^ promotes the development of hyper‐appetite and obesity in mice. Moreover, a series of studies have reported that the local release of Oxt is critical for regulating energy and body weight balance.^[^ ^36^ ^]^ HFD feeding markedly reduces Oxt release, and treatment of Oxt can effectively correct hyper‐appetite and obesity.^[^ ^22^, ^33^, ^37^ ^]^ Reduction of Oxt release by Oxt neuron ablation promotes appetite and decreases energy balance.^[^ ^22^, ^38^ ^]^ Oxt neuropeptide in the PVN is released via direct axonal connections to the critical brain regions linked to the control of energy balance, such as Arc, VMH, NTS, et al.^[^ ^38^, ^39^ ^]^ Clinical studies have revealed that Oxt nasal spray effectively induces metabolic improvements in patients with obesity.^[^ ^33^ ^]^ Herein, we found that Nrg4‐ErbB4 signaling evokes Oxt neurons and stimulates Oxt release. Interestingly, previous studies have shown that ErbB4 is localized at the neuron presynaptic terminals and is necessary for neurotransmitter release.^[^ ^10^, ^40^ ^]^ Nrgs‐ErbB4 also promotes the excitability of fast‐spiking neurons through ion channels regulation.^[^ ^32^, ^41^ ^]^ These studies indicated that ErbB4 promotes neuron excitability and neuropeptide release. Hence, the above evidence demonstrates that the released Oxt is a key mediator of the effects of Nrg4 on preserving metabolic homeostasis.

In conclusion, we demonstrated that peripheral Nrg4 can enter the hypothalamus and activate hypothalamic Oxt neurons. Central administration of rNrg4 ameliorates dietary obesity by reducing appetite and increasing energy expenditure. The Nrg4 receptor, ErbB4, is abundantly expressed in the hypothalamus, and its phosphorylation is reduced in HFD‐fed mice. ErbB4 in Oxt neurons plays a critical role in the regulation of metabolic homeostasis by regulating Oxt release. PVN Oxt is a critical mediator of the anti‐obesity effects of Nrg4. Collectively, our findings highlight central Nrg4‐ErbB4 signaling as a potential therapeutic strategy for the treatment of obesity and related diseases.

## 4 Experimental Section

### Animals

Male *C57BL/6* mice were purchased from Jicui Yaokang Biotechnology Co., Ltd.(Nanjing, China). *Oxt‐ires‐Cre*,^[^ ^42^ ^]^ *ErbB4‐2A‐CreERT2*,^[^ ^19^ ^]^ and *tdTomato reporter/Ai14* ^[^ ^21^ ^]^ mice were previously described. *ErbB4‐2A‐CreERT2* mice were crossed with *tdTomato/Ai14* mice to reporter ErbB4 expression. To induce genetic recombination in *ErbB4‐2A‐CreERT2* mice, tamoxifen was dissolved in corn oil and intraperitoneally injected for 5 days at a dose of 75 mg kg^−1^.^[^ ^21^ ^]^ All experimental procedures were approved by the Institutional Animal Care and Use Committee of Shanghai Sixth People's Hospital Affiliated to Shanghai Jiao Tong University School of Medicine (DWSY2022‐0542). Regular chow (9.4% kcal from fat) and HFD (60% kcal from fat) were purchased from Xietong Bioscience (Beijing, China) and Research Diets (New Brunswick, NJ, USA), respectively. The DIO mouse models were fed the HFD for 12 weeks starting at 6 weeks, unless otherwise noted.

### Protein Purification

In previous study, it was found that mutations in E47Q of Nrg4 enhanced the affinity of Nrg4 on binding to ErbB4.^[^ ^7b^ ^]^ Hence, the Nrg4 E47Q recombinant protein was used in this study. The Mouse Nrg4 (1‐61, E47Q) recombinant protein was expressed and purified using pET expression system (Novagen). Briefly, Mouse Nrg4 cDNA was PCR‐amplified and cloned into pET‐28a expression vector in frame with 6X histidine residues. The plasmid was transformed into *Escherichiacoli* Strain BL21‐Rosetta and cultured in lennox broth (LB) containing kanamycin (100 mg L^−1^). The BL21 strain was induced to produce histidine‐tag NRG4 by addition of 0.2 mm IPTG for 16 h at 16 °C. Lysates of BL21 strain was centrifuged, and the supernatant was collected and run through the Ni‐NTA agarose affinity column for recombinant Nrg4 purification. The Nrg4 proteins eluted from Ni‐NTA agarose column were pooled and dialyzed in saline buffers (PH = 7.4).

### Plasmid Construction and Virus Production

The Ctrl and Nrg4 expression plasmids were described previously.^[^ ^7b^ ^]^ To generate the ErbB4 expression lentivirus plasmid (ErbB4‐Lenti), mouse ErbB4 cDNA was cloned into the Ubi‐MCS‐3FLAG‐CBh‐gcGFP‐IRES‐puromycin vector at the BamHI and AgeI sites designed and packaged by Jikai Technology (Shanghai, China). To generate an Oxt neuron‐specific lentiviral vector (Oxt‐shCtrl), the hSyn promoter in the rLV‐hsyn‐mCherry‐5′miR‐30a‐shRNA(scramble)‐3′‐miR30a‐WPRE vector was replaced with the mouse Oxt promoter designed and constructed by BrainVTA Technology (Wuhan, China). The Oxt promoter sequence was >NC_000068.8:130416432‐130418974 Mus musculus strain C57BL/6J chromosome 2, GRCm39 from NCBI. The Oxt‐shErbB4 plasmid was constructed by inserting a short hairpin RNA (ShRNA) cassette for ErbB4 (5′‐CCAGA CTACC TGCAG GAATAC‐3)^[^ ^40a^ ^]^ to the Oxt‐shCtrl plasmid designed and constructed by BrainVTA Technology (Wuhan, China). Cre‐dependent AAV‐hSyn‐DIO‐hM4Di‐mCherry, AAV‐hSyn‐DIO‐hM3Dq‐mCherry, AAV‐hSyn‐DIO‐mCherry, and AAV‐EF1*α*‐DIO‐DTA were purchased from BrainVTA Technology (Wuhan, China).

### Surgery

For lateral ventricle cannulation, mice were anesthetized with avertin (300 mg kg^−1^) and placed in a stereotaxic instrument (RWD, Shenzhen, China). Next, a 28G guide cannula was implanted at the lateral ventricle, 0.6 mm posterior to the bregma, 2.0 mm below the skull surface, and 1 mm lateral to the bregma. For virus injection into the PVN, mice were anesthetized with avertin (300 mg kg^−1^) and placed in a stereotaxic instrument (RWD, Shenzhen, China). Using a micro syringe pump (KD Scientific, Holliston, USA), virus was bilaterally injected into the PVN (coordinates: A/P, −0.85 mm posterior to the bregma; M/L, ±0.2 mm; D/V, −4.8 mm). The mice were allowed to recover for 2 weeks before any experiments.

### Treatments

For rNrg4 treatment, DIO mice were implanted with a guide cannula targeting the lateral ventricle. After recovery, rNrg4 was administered daily at a dose of 500 ng immediately before the light was turned off. Food intake and body weight were measured daily (Figure S5D, Supporting Information). Cumulative food intakes were presented. To initially determine the i.c.v dose of rNrg4, 1, 10, 100, 500, or 1000 ng of rNrg4 were administered to the mouse brain and the food intake was measured in 8 h (Figure S3, Supporting Information). For administration of the AG‐1478 (MCE, HY‐13524), L‐372662 (MCE, L‐372662), Oxytocin (Sangon, A605015), and rNrg4 to DIO mice, lateral ventricle‐cannulated DIO mice were briefly fasted, and received i.c.v. administration of the ErbB4 antagonist AG‐1478 (20 nm,1 µL); the OTR antagonist, L‐372662 (2 µg µL^−1^, 1 µL); Oxytocin (1 µg µL^−1^, 1 µL); or 0.9% NaCl as a control. After 1 h and before lights were turned off, rNrg4 (500 ng µL^−1^, 1 µL) or NaCl was injected. Food intake and body weight were measured. For food intake measurement of ErbB4 CreER mice injected with AAV‐DIO‐mCherry, AAV‐DIO‐hM3Dq, and AAV‐DIO‐hM4Di viruses, mice under HFD feeding were briefly fasted and injected with CNO (MCE, HY‐17366) at the dose of 2 mg kg^−1^ before lights were turned off. For administration of rNrg4 on mice injected with AAV‐DIO‐mCherry, AAV‐DIO‐ hM3Dq, and AAV‐DIO‐hM4Di, mice were briefly fasted and injected with CNO and rNrg4, or 0.9% NaCl before lights were turned off.

### Measurement of Metabolic Parameters

Body composition of mouse was measured using DEXA (InAlyzer, Seoul, South Korea). Oxygen consumption, carbon dioxide, and heat production were detected using the Laboratory Animal Monitoring System (Columbus, St Paul, USA) and normalized by lean body mass. For central rNrg4 treatment, indirect calorimetry data included oxygen consumption (VO_2_), carbon dioxide production (VCO_2_), and energy expenditure (EE) for 0–8 h post‐infusion. For Oxt‐shCtrl and Oxt‐shErbB4 mice, the VO_2_, carbon VCO_2_, and EE were measured when mice were feeding HFD for 1 week after virus expression, at which time the body weight did not change (Figures S7C and S9E, Supporting Information). As per AAV‐GFP‐ and AAV‐DTA‐injected Oxt‐shCtrl and Oxt‐shErbB4 mice, rNrg4 was administered daily at a dose of 500 ng immediately before the lights were turned off. After which, VO_2_, VCO_2_, and EE were measured and analyzed in 12 h.

### Hematoxylin and Eosin Staining

The eWAT and liver tissues were fixed in 4% paraformaldehyde (PFA) and embedded in paraffin. Tissues were sectioned at a thickness of 5 µm and stained with H&E solution. Images were obtained using a Leica DM4 B microscope (Leica, Buffalo Grove, USA). Areas of eWAT were analyzed using Image‐Pro Plus (Ver 6, Media Cybernetics, Rockville, MD, USA).

### Glucose Tolerance Test and Serum TG Analysis

For the GTT, mice were fasted for 12–16 h and intraperitoneally injected with D‐glucose solutions (1.5 g kg^−1^ body weight). Blood glucose levels were measured at 0, 15, 30, 60, 90 and 120 min using a glucometer (Roche, Basel, Switzerland). For GTT following central rNrg4 treatment, mice were tested 2 h after i.c.v. administration of rNrg4. Serum TG levels were assayed with reagents from Bayer using an automatic biochemical analyzer.

### Immunofluorescence

Immunofluorescence analysis was performed as previously described.^[^ ^43^ ^]^ Briefly, mice were anesthetized with avertin (300 mg kg^−1^) and fixed with pre‐cooled 4% PFA through transcardial perfusion. Brain tissue was post‐fixed in 4% PFA at 4 °C for 4 h and subjected to gradient dehydration in 20% and 30% sucrose solutions at 4 °C overnight. Tissues were sectioned at a thickness of 25 µm using a cryostat (CM1950; Leica). Sections were then blocked with 5% serum diluted with 0.3% Triton X‐100/PBS, incubated with rabbit anti‐ErbB4 (1:100, Proteintech, 19943‐1‐AP), rabbit anti‐c‐Fos (1:1000, CST, #2250), guinea pig anti‐c‐Fos (1:2000, sysy, 2 26 017), rabbit anti‐Oxt (1:500, Immunostar, 20 068), and rabbit anti‐AVP (1:500, Abcam, ab213708) primary antibodies at 4 °C overnight, followed by incubation with fluorescently labeled secondary antibodies for 1 h at room temperature. The slides were incubated with DAPI to visualize cell nuclei. Images were acquired using an LSM980 confocal microscope (Carl Zeiss, Jena, Germany) and analyzed using ImageJ (Ver. 1.8, NIH, Bethesda, MD). For double immunofluorescence of ErbB4 and PVN neurons, slides of ErbB4‐CreER::Ai14 mice were incubated with green fluorescent Nissl stain (1:2000, Thermofisher, N21480) for 5 min at room temperature. For specificity of ErbB4 antibody verification, Neuro2a cells were transfected with Lenti‐GFP vector expressing Flag‐tagged ErbB4 or empty vector for 24 h. Double immunofluorescence staining of ErbB4 and Flag were performed on fixed cells. For each mouse, cells were counted manually on one side of the hypothalamus in representative images.

### Quantitative RT‐PCR

Total RNA was extracted using TRIzol reagent (Thermo Fisher). mRNA was reverse‐transcribed to cDNA using an RT reagent kit (Takara). RT‐PCR was performed using SYBR Green Premix (Thermo Fisher) on a QuantStudio 7 Flex Real‐Time PCR System (Thermo Fisher). The 2^−ΔCt^ method was used to determine relative mRNA levels, where ΔCt is the difference between the Ct value of the target gene and GAPDH control. Table S1, Supporting Information presents the primer sequences.

### Western Blot

Total proteins were extracted using tissue lysis buffer containing protease and phosphatase inhibitors. Protein levels were determined using a BCA protein concentration assay kit. Protein samples were separated using SDS‐PAGE and transferred to PVDF membranes (Millipore). Membranes were blocked with 5% non‐fat milk (CST) and incubated with rabbit anti‐ErbB4 (1:1000, CST, #4795), rabbit anti‐His (1:1000, CST, #12 698), rabbit anti‐pErbB4 (1:100, CST, #4757), rabbit anti‐HSP90 (1:1000, CST, #4874), rabbit anti‐GAPDH (1:1000, CST, #5174), and rabbit anti‐*β*‐actin (1:2000, CST, #4967) antibodies.

### Oxt Release Assay

The Oxt release assay was performed as described previously.^[^ ^22^, ^44^ ^]^ To measure the effect of Nrg4 on Oxt release, tissue slices containing the PVN from DIO mice were sectioned into small pieces of ≈0.5 mm and immediately balanced in Locke's solution supplemented with 95% O_2_ and 5% CO_2_ at 37 °C. The solution was changed every 5 min for a total of five times during the experimental period, and the fifth sample was collected to measure the basal release rate. PVN tissues were then incubated in the same solution containing Nrg4 (1 µg mL^−1^) for 5 min, and the solution was measured to detect Nrg4‐evoked Oxt release. To examine the effect of ErbB4 overexpression in the PVN on Oxt release, Ctrl‐L and ErbB4‐L viruses were injected into the PVN of HFD‐fed mice. PVN‐containing slices were sectioned into small pieces of ≈0.5 mm and immediately balanced in Locke's solution supplied with 95% O_2_ and 5% CO_2_ at 37 °C. The solution was changed every 5 min for a total of five times during the experimental period, and the fifth sample was collected to measure the release rate. To demonstrate the effect of ErbB4 inhibition of Oxt‐expressing neurons on Oxt release, the PVN slices of Oxt‐shCtrl and Oxt‐shErbB4 were sectioned into small pieces of ≈0.5 mm and immediately balanced in Locke's solution supplied with 95% O_2_ and 5% CO_2_ at 37 °C. The solution was changed every 5 min for a total of five times during the experimental period, and the fifth sample was collected to measure the basal release rate. Oxt concentrations were measured using an EIA kit (Enzo, Farmingdale, NY, USA). For Serum Oxt levels assay, Oxt‐shCtrl or Oxt‐shErbB4 mice fed a HFD were injected with saline (control) or rNrg4. The serum was collected from the mice accordingly.

### Statistical Analysis

All data are presented as mean ± SEM. Data were analyzed using Prism 9 (GraphPad Software, San Diego, CA, USA). A two‐tailed Student's *t*‐test was used for two‐group comparisons. One‐way and two‐way analysis of variance (ANOVA) with Bonferroni's post hoc test were used for comparisons of more than two groups. *p*‐values < 0.05 were considered statistically significant.

## Conflict of Interest

The authors declare no conflict of interest.

## Author Contributions

Y.Z., Y.Z., J.W., L.J., and M.G. contributed equally to this work. Y.Z. designed and performed the experiments, analyzed the data, and wrote the manuscript. Y.Z., Y.Z., J.W., L.J., M.G., L.C., L.Z., and B.W. performed the experiments and analyzed the data. Y.L. helped to design the project. R.Z. provided valuable advice on the project. W.J. provided advice and participated in discussions throughout this work. C.H. conceived the study, designed the experiments, and revised the manuscript. All authors read and approved the final version of the paper.

## Supporting information

Supporting Information

## Data Availability

The data that support the findings of this study are available from the corresponding author upon reasonable request.
