# Supplementary material for: Neuregulin4 Acts on Hypothalamic ErBb4 to Excite Oxytocin Neurons and Preserve Metabolic Homeostasis
Source: Adv Sci (Weinh). 2023 Apr 14;10(16):2204824. doi: 10.1002/advs.202204824 (PMC10238187; doi:10.1002/advs.202204824)
Supplement: Supplementary file 1 — Supporting Information [file ADVS-10-2204824-s001.pdf]

Supporting Information

Neuregulin4 Acts on Hypothalamic ErBb4 to Excite Oxytocin Neurons and Preserve Metabolic Homeostasis

Yi Zhang, Yangyang Zhu, Jinghui Wang, Li Jin, Mingwei Guo, Liwei Chen, Lina Zhang, Yangyang Li, Baocheng Wan, Rong Zhang, Weiping Jia, Cheng Hu*

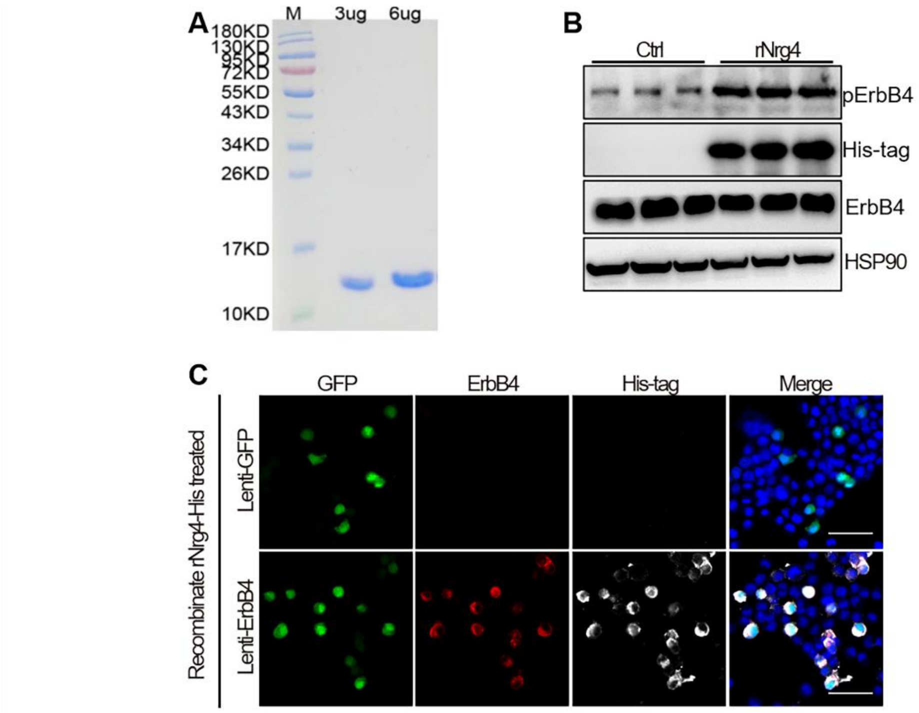

Figure S1. Purification and verification of Nrg4 recombinant protein. A) Coomassie brilliant blue staining of rNrg4. B) HEK293 cell were treated with rNrg4 (20 μg/mL) for 20 min, then WB for pErbB4, His-tag, and ErbB4 was performed. HSP90 was used as loading control. C) Neuro2a cells were transfected with Lenti-GFP or Lenti-ErbB4, and then treated with rNrg4-His (20 μg/mL) for 2 h. Double immunofluorescence staining of ErbB4 (red) and His (grey) was performed. Cell nuclei were counterstained with DAPI (blue). Scale bar, 50 μm.

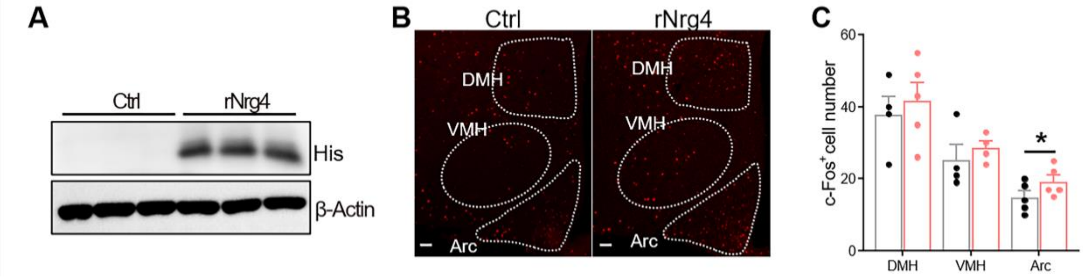

Figure S2. Characterization of the mice intraperitoneal injection with Saline or rNrg4**.** A) Immunoblots of hypothalamic lysates from mice intraperitoneal injection with Saline or rNrg4 (500 μg/kg). B) Representative immunofluorescence staining of c-Fos in the DMH, VMH, and Arc nuclei of mice intraperitoneally injected with rNrg4 (500 μg/kg) or control. Scale bar, 50 μm. (C) Numbers of c-Fos$^+$ cells in the DMH, VMH, and ARC (DMH: n = 4 for Ctrl, n = 5 for rNrg4; VMH: n = 4; Arc: n = 5). Data are presented as mean ± SEM; *$p < 0.05$, two-tailed Student's t-test (C).

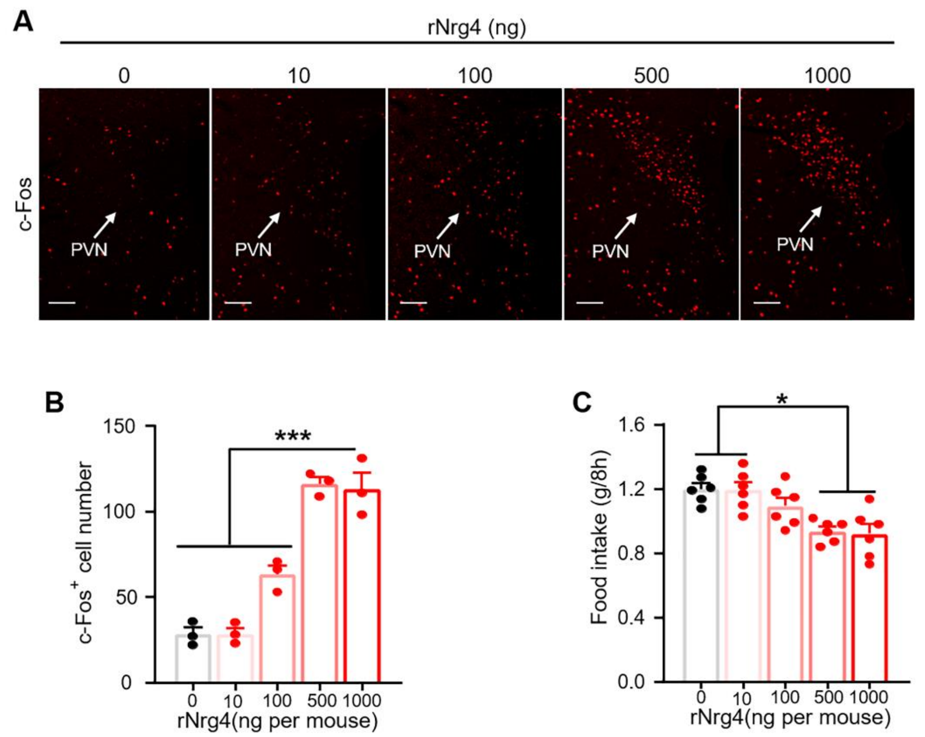

Figure S3. Determine the i. c. v. injection dose of rNgr4. A) Mice were i. c. v. administered different doses of rNrg4. Then, mice were heart perfused after injection for 2 h. Representative immunofluorescence staining of c-Fos in the PVN of mice. Scale bar, 50 μm. B) Numbers of c-Fos$^+$ cells of mice receiving different doses of rNrg4 injection (n = 3 for each group). Data are presented as mean ± SEM; *p < 0.05 and ***p < 0.001, two-way analysis of variance (ANOVA) with Bonferroni's post hoc test (B, C).

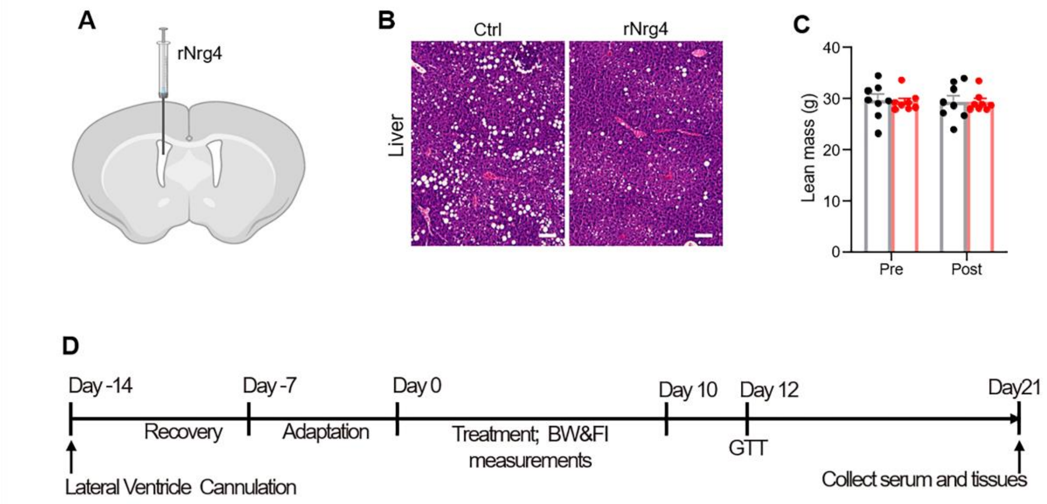

Figure S4. Nrg4 targets the brain to preserve metabolic homeostasis. A) Schematic diagram of lateral ventricular injections of rNrg4. B) H&E staining of liver from mice administered Ctrl or rNrg4. Scale bar, 100 μm. C) Lean mass before and after treatment with Ctrl or rNrg4 (*n* = 8 for each group). D) Schematic diagram of the experimental procedures for central treatment with rNrg4.

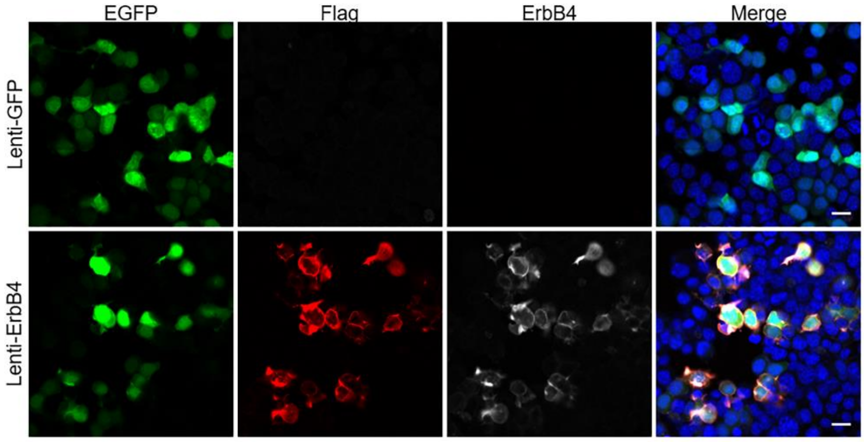

Figure S5. Verification of the specificity of the ErbB4 antibody. Neuro2a cells were transfected with Lenti-GFP vector expressing Flag-tagged ErbB4 or empty vector. Double immunofluorescence staining of ErbB4 (Red) and Flag (Gray) was performed on fixed cells. Cell nuclei were counterstained with DAPI (blue). Scale bar, 20 um.

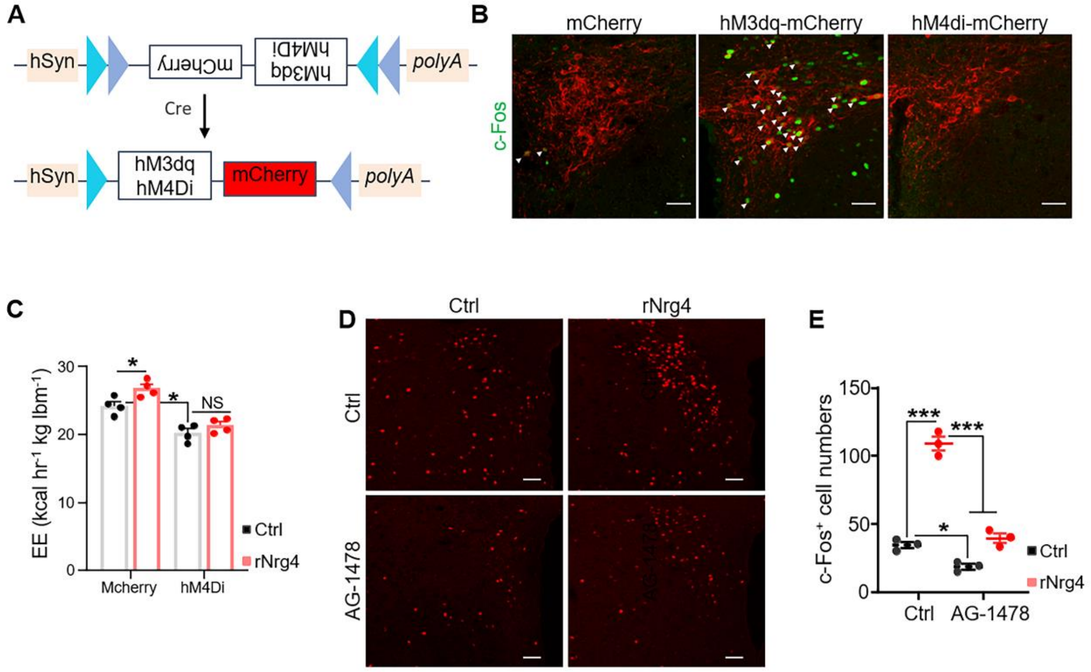

Figure S6. PVN ErbB4 neurons mediate Nrg4's effect on energy balance. A) Diagram of Cre-dependent expression of mCherry, hM3Dq-mCherry and hM4Di-mCherry. B) *ErbB4 CreER* mice expressed mCherry, hM3Dq-mCherry and hM4Di-mCherry in PVN ErbB4 cells were treated with CNO and sacrificed two hours after the treatment. Brain sections were immunostaining with c-Fos in the PVN. White arrows indicate cells that express both c-Fos and mCherry. Scale bar, 50 µm. D) *ErbB4 CreER* mice expressed mCherry and hM4Di-mCherry in PVN were treated with saline or rNrg4. EE for 4-h posttreatment is shown (n = 4 for each group). lbm, lean body mass. E) Immunofluorescence staining of c-Fos in the PVN of mice treated with rNrg4 and AG-1478. Scale bar, 50 µm. F) Numbers of c-Fos^{+} cells in the (n = 3 for each group). Data are presented as Mean ± SEM; *p < 0.05, **p < 0.01, ***p < 0.001, two-way analysis of variance (ANOVA) with Bonferroni's correction (C, E).

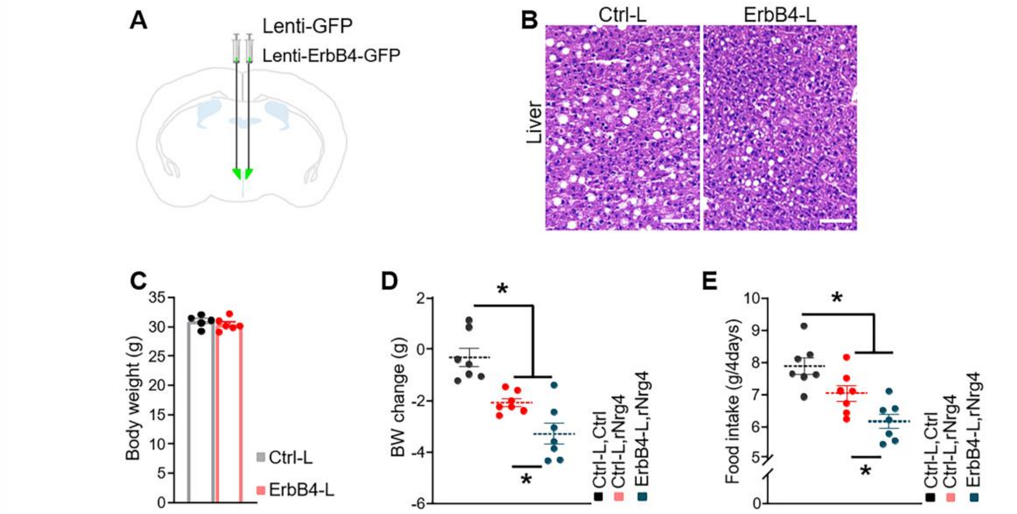

Figure S7. Overexpression of ErbB4 in the PVN protects against DIO. A) Schematic diagram depicting intra-PVN injections of Ctrl-L and ErbB4-L viruses in mice. B) Liver H&E staining of Ctrl-L and ErbB4-L mice fed a HFD for 20 weeks. Scale bar, 100 μm. C) Body weights of Ctrl-L and ErbB4-L mice used for indirect calorimetry analysis. D, E) HFD-fed mice were injected with Ctrl-L or ErbB4 virus into the PVN. A cannula targeting the third ventricle was implanted. Mice received i.c.v. injections of control or 500 ng of rNrg4 daily for 4 days. Body weight changes (D) and cumulative food intake(E) were then measured (n = 7 for each group). Data are presented as Mean ± SEM; *$p < 0.05$, one-way analysis of variance (ANOVA) with Bonferroni's correction (D, E).

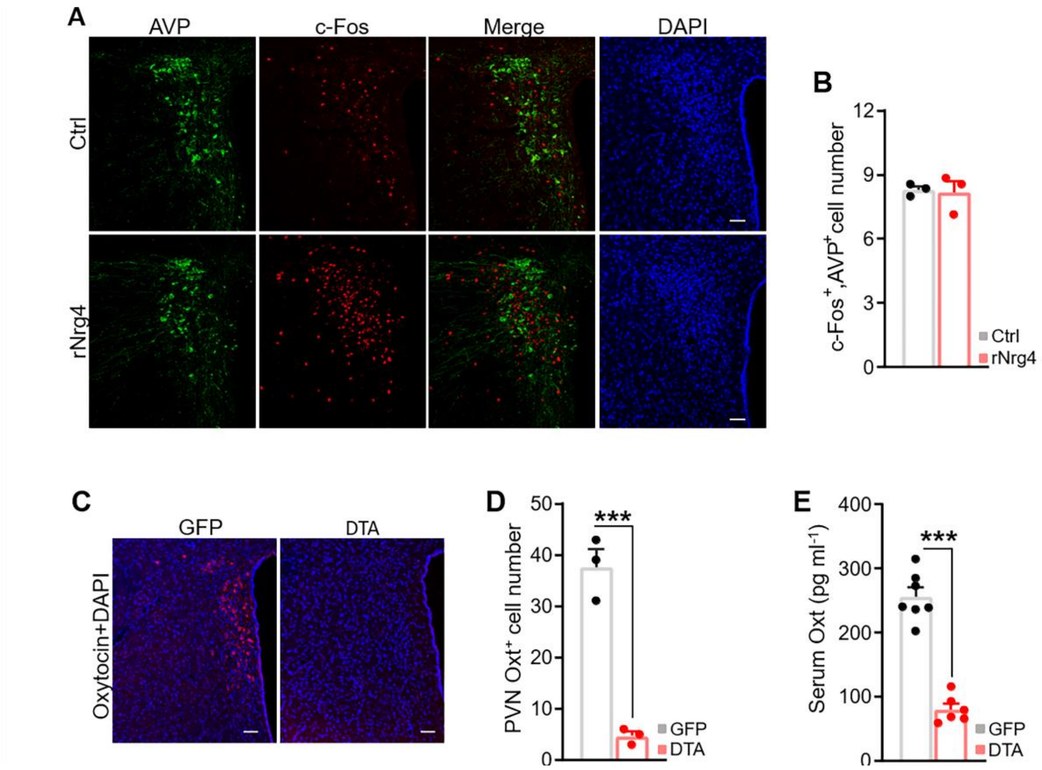

Figure S8. A) Double immunofluorescence staining of vasopressin (AVP, green) and c-Fos (red) in the PVN of mice administered control or rNrg4. Cell nuclei were counterstained with DAPI (blue). Scale bar, 50 μm. B) Number of c-Fos$^+$ and AVP$^+$ cell number in the PVN (n = 3 for each group). Scale bar, 50 μm. C) Presentative images of PVN Oxt expression in *Oxt-ires-Cre* mice injected with AAV-DIO-GFP and AAV-DIO-DTA viruses for 10 weeks. Scale bar, 50 μm. D) Number of Oxt+ cells in the PVN (*n* = 3 for each group). E) The serum Oxt levels in *Oxt-ires-Cre* mice injected with AAV-DIO-GFP and AAV-DIO-DTA viruses for 8 weeks (*n* = 7 for AAV-DIO-GFP, *n* = 6 for AAV-DIO-DTA). Data are presented as mean ± SEM; ****p* < 0.001, two-tailed Student's t-test (D, E).

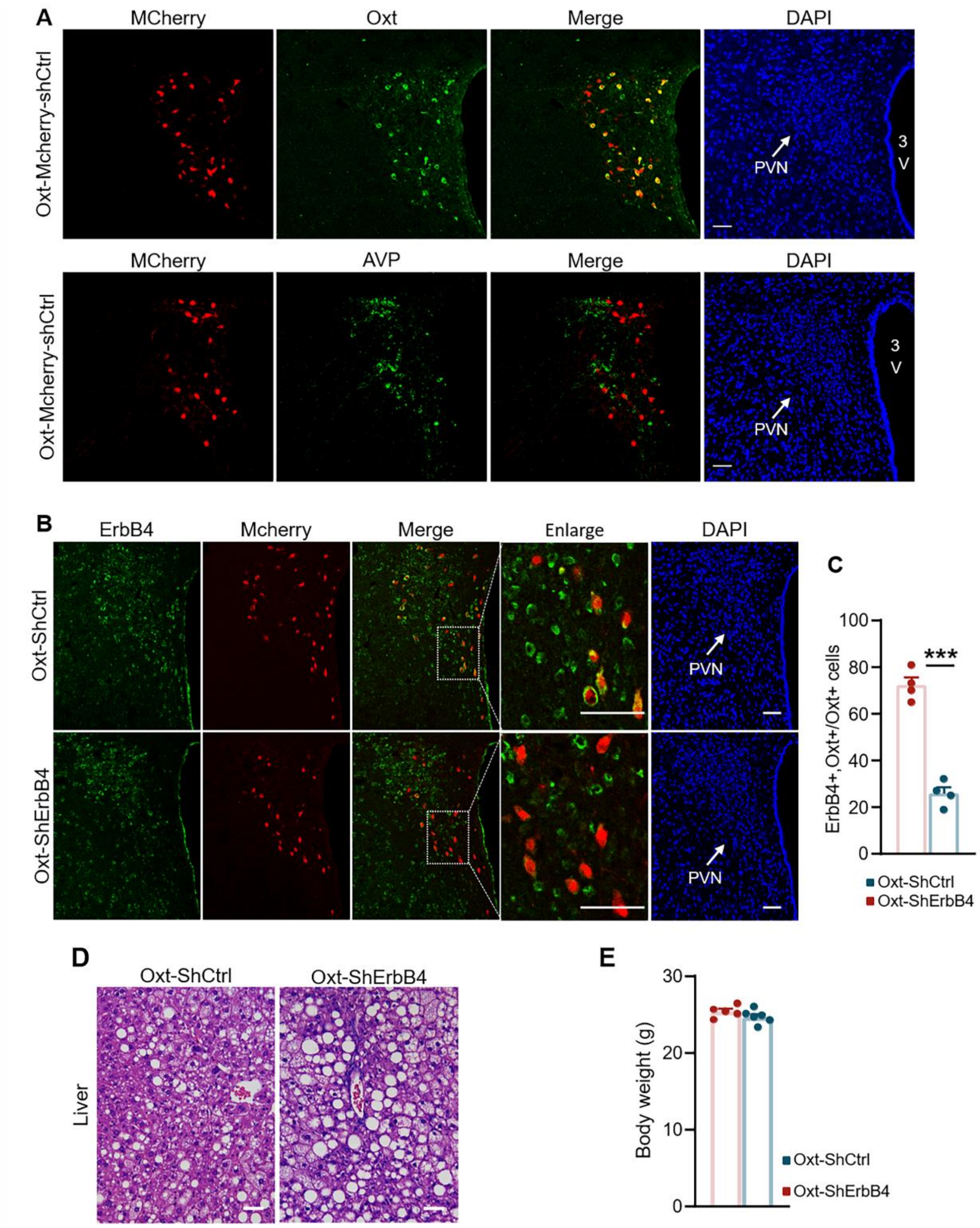

Figure S9. Characterization of the Oxt-shCtrl and Oxt-shErbB4 mice. A) Mice were intra-PVN injected with Oxt-MCherry-shCtrl, and immunofluorescence staining of Oxt and AVP was performed. Cell nuclei were counterstained with DAPI (blue). Scale bar, 50 µm. B) Immunofluorescence staining of ErbB4 in the PVN of mice microinjected with Oxt-shCtrl or Oxt-shErbB4. Cell nuclei were counterstained with DAPI (blue). Scale bar, 50 µm. C) Quantification of $ErbB4^+$, $mCherry^+/ErbB4^+$ cells in Oxt-shCtrl and Oxt-shErbB4 mice in mCherry (Oxt) neurons (n = 4 for each group). D) H&E staining of Oxt-shCtrl and Oxt-shErbB4 mice fed a HFD for 12 weeks. Scale bar, 100 µm. E) Body weights of Oxt-shCtrl and Oxt-shErbB4 fed a HFD for 1 weeks, used for indirect calorimetry analysis (n = 5 for Oxt-shCtrl, n = 6 for shErbB4). Data are presented as mean ± SEM; ****p* < 0.001, two-tailed Student's t-test (**C**).

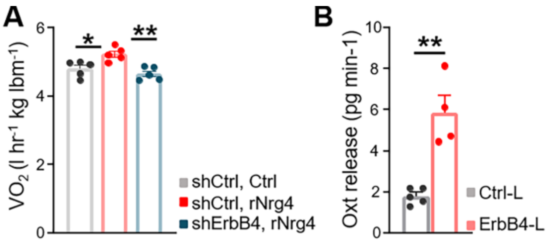

Figure S10. A) Oxt-shCtrl or Oxt-shErbB4 mice under a HFD fed were treated with rNrg4 or saline, 0-12 h oxygen consumption (VO_2) in the dark cycle was measured (*n* = 5 for each group). (B) Basal Oxt release from PVN slices of Ctrl-L and ErbB4-L mice (*n* = 6 for Ctrl-L, *n* = 4 for ErbB4-L). Data are presented as mean ± SEM; **p* < 0.05, ***p* < 0.01, one-way analysis of variance (ANOVA) with Bonferroni's correction (A), two-tailed Student's t-test (B).

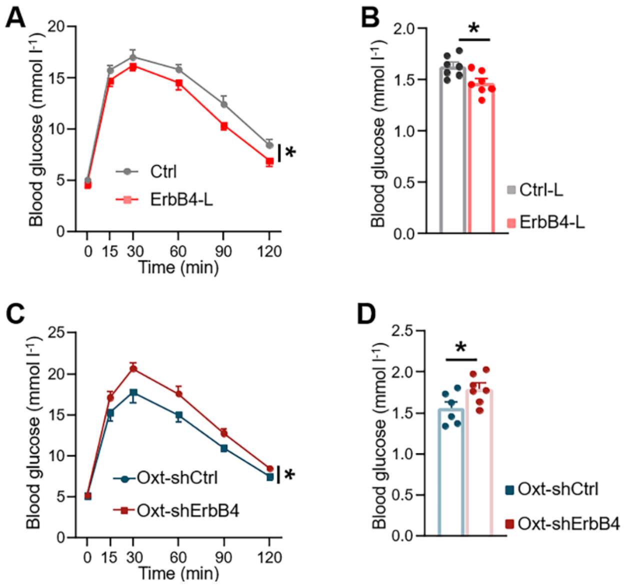

Figure S11. A, B) GTT and AUC of GTT of Ctrl-L and ErbB4-L mice fed a HFD for 4 weeks (*n* = 7 for each group). C, D) GTT and AUC of GTT of Oxt-shCtrl and Oxt-shErbB4 mice fed a HFD for 4 weeks (*n* = 6 for Oxt-shCtrl, *n* = 7 for Oxt-shErbB4). Data are presented as mean ± SEM; *$p < 0.05$, two-way analysis of variance (ANOVA) with Bonferroni's correction (A, C), two-tailed Student's t-test (B, D).

Table S1. Primer sequences

**Gene**	**Primer**	**Sequence (5'->3')**
Nrg4	Forward	CCCAGCCCATTCTGTAGGTG
	Reverse	ACCACGAAAGCTGCCGACAG
ErbB4	Forward	ACCTCAACACCTTCGCCAATGC
	Reverse	GGCAGGCTGTGGTTCCAGTAGT
Gapdh	Forward	CCCACTCTTCCACCTTCGAT
	Reverse	CCTCTCTTGCTCAGTGTCCT
